# Data on early assessment of knowledge, attitudes, and behavioral responses to COVID-19 among Connecticut residents

**DOI:** 10.1016/j.dib.2020.106347

**Published:** 2020-09-24

**Authors:** Toan H. Ha, Stephen L. Schensul

**Affiliations:** a Department of Infectious Diseases and Microbiology, Graduate School of Public Health, University of Pittsburgh, 130 DeDoto Street, Pittsburgh, PA 15261, USA; b Department of Public Health Sciences, School of Medicine, University of Connecticut, Farmington, CT, USA

**Keywords:** COVID-19, Knowledge, Perceived risk, Perceived seriousness, Anxiety, Adoption of prevention behaviors, Information, Connecticut

## Abstract

The survey dataset presented in this article examines COVID-19-related knowledge, attitudes, perceived risk and adoption of prevention behaviors. The survey was conducted anonymously among non-random sample of 464 Connecticut residents in the early stage of social distancing and shutdown from March 23 to March 29, 2020. The questionnaires included five major groups of questions. 1) Demographic information 2). Perceived risk, perceived seriousness and anxiety related to COVID-19; 3). Knowledge of COVID-19, adoption of preventive behaviors and health seeking behaviors; 4). Duration of accumulating of food, household supplies and medicine stockpiling for possible shortage; 5). Sources of information about COVID-19. Data were analyzed using frequencies, percentages, means, and standard deviations. The data provides neccessary evidence to develop effective communication messages and prevention strategy to address the COVID-19 and future pandemic.

## Specification Table

**Table:** 

Subject	Infectious diseases and public health
Specific subject area	Health behaviours and behavioral sciences
Type of data	Table, Figure
How data were acquired	Data were collected via online survey using a Qualtrics hyperlink and converted in SPSS version.26 for analysis. A copy of the survey is included as supplementary file.
Data format	Raw analyzed
Parameters for data collection	The target population of the survey was non-healthcare related participants. The survey assessed participants’ knowledge, perceived risk, perceived seriousness, anxiety, adoption of prevention behaviors and source of information related to COVID-19. The questionnaire also collected respondents’ demographic characteristics and occupation.
Description of data collection	Data were collected anonymously from a non- random sample of 464 Connecticut residents. Participants were contacted by e-mail, phone, skype or other communication means. Participants could choose to use the Qualtrics hyperlink for self-administration of the survey online or recruiters could administer the survey as an interview over the phone or other means of communication and record their answers online.
Data source location	Institution: University of Connecticut School of Medicine City/Town/Region: Farmington, Connecticut Country: United States.
Data accessibility	Data is uploaded on Mendeley Repository: http://dx.doi.org/10.17632/zjydvt8sk9.1

## Value of the Data

• This dataset are useful as it provides real time information of public reaction to COVID-19 in the early stage of social isolation in the US.
• Public health agencies can use the data to identify gaps in effective communication and develop more effective public health messages on COVID-19.
• The data can be used to compare with other countries regarding early public responses to COVID-19 pandemic.
• Data provide further insights about participants’ mental responses to COVID-19 which can be used to develop effective messages to reduce fear and anxiety in the community.
• The data can be further analyzed to assist in development of public health advocacy and interventions to help prevent the future pandemic.

## 1 Data Description

**Fig. 1:**
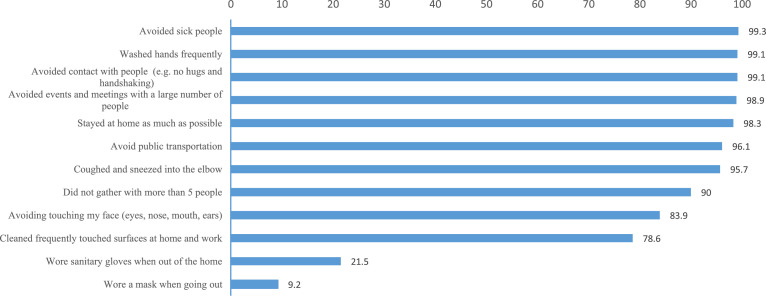
Proportion of adoption of prevention behaviors to COVID-19.

The US has the highest number of Covid-19 infection cases and has the highest COVID-19 related death [1]. Connecticut is one of the US States that has been hit hard by the COVID-19 pandemic [2]. This dataset generated from a survey assessing early public response to COVID-19 among 464 Connecticut residents conducted in the early stages of social distancing and shutdown. The dataset included five major groups of variables. 1) Demographic information including age, education, gender, marital status, employment, and ethnicity; 2). Participants’ perceived risk, perceived seriousness and anxiety related to COVID-19; 3). Participants’ knowledge of COVID-19, adoption of preventive behaviors and health seeking behaviors when having coronavirus-like symptoms; 4). Duration of accumulating of food, household supplies and medicine stockpiling for possible shortage; 5). Sources of information from which participants received information about COVID-19. Detailed descriptions of all variables, together with the questions for each variable, and descriptive tables and figures can be found in the Mendeley data repository [3] and Tables 1–4 and Fig. 1.

**Table 1:** Descriptive statistics of sample characteristics (*n* = 464).

Variables	Categories	Frequency	Percent	Statistics
**Age**	19–30	128	37.0	Mean =41.1 (S*D* = 14.3)
	31–59	173	50.0	
	Over 60	45	13.0	
**Gender**	Male	135	29.1	
	Female	323	70.5	
	Missing	6	1.3	
**Education**	<=High school	29	6.3	Mean =15.9 (SD =4.5)
	Some college	59	12.7	
	=>Bachelor's degree	305	65.7	
	Missing	71	15.3	
**Marital Status**	Single, never married	117	25.2	
	Married or living with partner	284	61.2	
	Divorced or Separated	42	9.1	
	Widowed	12	2.6	
	Missing	9	1.9	
**Ethnicity**	African American	16	3.4	
	Latino/Hispanic	16	3.4	
	White	252	54.3	
	American Indian	4	0.9	
	Asian	14	3.1	
	Other	147	32.7	
	Missing	15	3.2	
**Occupation**	Professional	162	34.9	
	Salaried	118	25.4	
	Hourly	102	22.0	
	House/Husband or Wife	33	7.1	
	Students	30	6.5	
	Missing	19	4.1

**Table 2:** Perceived risk, perceived seriousness and anxiety related to COVID-19 (*n* = 464).

**Variable**	Responses (%)					Mean (SD)
**Perceived risk**	Not serious 1	Less serious 2	Neutral 3	Serious 4	Very serious 5	
	23 (5.0)	82 (17.7)	193 (41.6)	116 (25.0)	48 (10.3)	3.2 (1.0)
**Perceived seriousness**	Not likely 1	Less likely 2	Neutral 3	Likely 4	Very likely 5	
	23 (5.0)	49 (10.6)	103 (22.2)	22.2	39.9	3.8 (1.2)
**Anxiety**	Very low 1	Low 2	Neutral 3	High 4	Very high 5	
	18 (3.9)	68 (14.7)	155 (33.4)	157 (33.8)	48 (10.3)	3.4 (1.0)

**Table 3:** COVID-19-related knowledge (Mean= 4.2, S.D. = 0.76).

Number of correct answers	Frequency	Percent
None	1	0.2
One	3	0.6
Two	6	1.3
Three	55	11.9
Four	247	53.2
Five	152	32.8

**Table 4:** Source of information about COVID-19 (*n* = 464).

Sources of information	Frequency	Percent
Electronic media and TV	392	85.0
Social media	371	81.4
Printed media	186	41.3
Healthcare provider/hospital	280	61.9
Family members	310	68.6
Friends	309	68.7
Clergy	38	8.2

## 2 Survey Design, Materials and Methods

This cross-sectional anonymously survey using a non-random approach was conducted to assess COVID-19 related knowledge, attitudes, and prevention behaviors among 464 Connecticut residents conducted in the early stages of social distancing and social isolation on March 23, one week after a stringent stay-at-home directive was implemented in Connecticut and closed to recruitment on March 29, 2020. Since the survey was conducted by the time when a social isolation and shutdown were just initiated in the State and only virtual meetings were allowed, we relied on faculty and students in the Department of Public Health Sciences to recruit participants for the study. They were asked to identify 3–5 non-healthcare-related people in their social network, but outside of their family or kin. Participants were contacted (e-mail, phone, skype or other means) by the faculty and students, and the telephone consent script was read to them. If consent was obtained, participants could choose to use an IP address for the survey and complete it online or faculty or students could administer the survey on the phone or other means of communication and record their answers online. The questionnaire was developed using Qualtrics software. [4] (See Appendix). The study was approved by University of Connecticut School of Medicine Institutional Review Board.

The survey is composed of five parts. The first part assessed demographic information including age, education, gender, marital status, employment, and ethnicity; 2). The second part included questions about perceived risk, perceived seriousness and anxiety related to COVID-19 in which respondents were asked questions regarding self-perception of the seriousness of COVID-19 for themselves with a 5-point Likert scale ranging from “Not likely” to “Very likely”. One question asked the perceived risk of acquiring disease with a 5-point Likert scale ranging from “Not likely” to “Very likely”. One question asked the anxiety level associated with COVID-19 with a 5-point Likert scale ranging from “Very low” to “Very high”; 3). The third part included questions about knowledge of COVID-19 and adoption of preventive behaviors in which respondents’ knowledge of COVID-19 was assessed with nine True/False questions. Adoption of Preventive behaviors to COVID-19 were assessed with 12 Yes/No questions. Health seeking behaviors were assessed using 16 True/False questions; 4). The fourth part included questions about duration of food, household supplies and medicine stockpiling using a five Yes/No questions asking participants about the essential items they accumulated in preparation for possible shortages in the following few weeks. The final part included Seven Yes/No questions asking about all sources of information from which participants received information about COVID-19. Data was transferred from Qualtrics to SPSS Version 26.0 for analysis. Data were analyzed using frequencies, percentages, means, and standard deviations.

## Ethics Statement

Informed consent was obtained from all participants before the survey was administered. Participants were informed that the survey was anonymous and that their participation in the survey was completely voluntary.

## Declaration of Competing Interest

This study did not receive any specific grant from funding agencies in the public, commercial, or not-for-profit sectors. The authors reported no conflicts of interest and have no competing financial interests or personal relationships that could have appeared to influence the work reported in this paper.

## Appendix Supplementary materials

Image, application 1
